# List of Cases Treated in the Surgical Wards of the Pennsylvania Hospital, and Discharged between November 1st and 15th, 1838

**Published:** 1838-11-21

**Authors:** Henry H. Smith

**Affiliations:** Resident Surgeon


### CLINICAL REPORTS.

### PENNSYLVANIA HOSPITAL.

*List of Cases treated in the Surgical Wards of the Pennsylvania Hospital, and discharged between November 1st and 15th,* 1838. Dr. T. Harris, Attending Surgeon.

[Reported by Henry H. Smith, M.D., Resident Surgeon.]

A case of reducible inguinal hernia of the right side was admitted November 2d; reduced immediately by taxis; truss applied, and the patient discharged the same day.

A case of severe contused wound of the back of the hand, in a girl of twelve years of age, caused by the hand being drawn in between two cog wheels, was admitted on the 15th of October. The integuments over the metacarpal bones were lacerated for about two inches, but there was no fracture. The hand and arm were loosely bandaged on a splint, and lead water applied. This dressing was continued for seven days, and prevented any sloughing of the contused part. The wound afterwards was treated as a simple ulcer, and the patient was discharged cured on the 3d of November, with the perfect use of the hand.

A case of severly lacerated wound of the hand, in a young man of eighteen years of age, caused by a load of shot passing through the second and third metacarpal bones, separating them nearly to the wrist, was admitted on the 9th of October. The case did remarkably well, and the patient was discharged on November 7th, with nearly the perfect use of his hand. The treatment will be reported hereafter.

A case of severe burn of the whole body and extremities, of a child of eleven years of age, caused by the clothes taking fire, was admitted on November 4th. The patient was wrapt in raw cotton; an anodyne given, and warmth applied to the extremities; delerium ensued, and death within twenty-four hours.

A case of lacerated wound of the fleshy part of the forearm of a man, caused by the bite of a dog, four days previous to his entrance, was admitted on November 1st. The wound was poulticed, placed on a splint, and treated, as a simple ulcer, by perfect rest. The wound healed, and the man was discharged six days after admission.

A case of sprained ankle, in a black woman, caused by a slip from a step, was admitted October 8th. There was considerable heat and excessive pain about the joint. The limb was placed at rest in a fracture box; lead water applied; and the patient not allowed to move until five days before her discharge, when a firm bandage was applied, and motion permitted. Discharged November 7th, thirty days after admission.

A second case of sprain of the ankle-joint in a man, caused by a twist of the foot in walking, was admitted October 17th. There was great effusion and heat about the joint. The limb was placed at rest, cold applications made to it constantly, and four dozen leeches applied in the evening. The same course, as to motion, was pursued in this case, and the patient discharged cured, November 14th,—one week after he began to use his crutches, and twenty eight days after admission.

A case of fracture of the neck of the thigh-bone, in a man sixty-six years of age, caused by a fall, was admitted April 2d, 1838, four days after the accident. He had been treated in the city for rheumatism, and the fracture was not detected until the day before his admission. The joint was much enlarged, and the limb shortened about an inch and a half. The patient was placed in bed, and Desault's apparatus applied for a week, with a view of bringing the limb to its original length, and keeping it more entirely at rest. Sloughing of the heel, and also of the outer side of the foot, followed, caused by the extending band, and the air of the ward, which, at that time, caused all the wounds to take on a bad appearance. The limb was then placed loose on a double inclined plane; stimulating and common poultices applied, with occasionally a wash, composed of fifty drops of pure nitric acid to the pint of water. The ulcers did not heal until the last of September, and the patient was confined to bed until October 14th, when he was allowed to use crutches. Liniments, &c., were afterwards used, and a firm bandage applied all the way up. Discharged on the 10th of November; limb partially useful, but considerably shortened.

A case of extensive wound of the upper part of the thigh, caused by a blow from a large piece of stone, which was thrown through the window of a house in the neighbourhood of a quarry, was admitted October 12th; treated by poultices and rest; after the slough separated, dressed as a simple ulcer, and discharged cured, November 7th, twenty-six days after admission.

A case of fracture of both bones of the leg, was admitted October 2d; treated in the usual way, and discharged at the request of the patient, the bones being quite firm, November 10th, forty days after the accident.

The case of fracture of the thigh, mentioned above, occurred in an old drunkard formerly in the alms-house. The extension made was slight, and would, in most instances, have been borne without injury, but owing to the bad constitution of the patient, and the tendency to hospital gangrene, prevalent at the time, it caused an exceedingly troublesome, ill-conditioned sore, the discharge and irritation of which strongly affected the patient. All attempts, therefore, to treat the fracture were given up, the limb was placed in any manner most comfortable to the patient, and the main part of the treatment directed to the ulceration.

